# Nanocomposite of Nickel Nanoparticles-Impregnated Biochar from Palm Leaves as Highly Active and Magnetic Photocatalyst for Methyl Violet Photocatalytic Oxidation

**DOI:** 10.3390/molecules27206871

**Published:** 2022-10-13

**Authors:** Is Fatimah, Hiroko Kawaii Wijayanti, Galih Dwiki Ramanda, Muchammad Tamyiz, Ruey-an Doong, Suresh Sagadevan

**Affiliations:** 1 Department of Chemistry, Faculty of Mathematics and Natural Sciences, Universitas Islam Indonesia, Kampus Terpadu UII, Jl. Kaliurang Km 14, Sleman, Yogyakarta 55584, Indonesia; 2 Universitas Nahdlatul Ulama Sidoarjo, Jl. Lingkar Timur KM 5, 5 Rangkah Kidul, Kecamatan Sidoarjo, Sidoarjo 61234, Indonesia; 3 Institute of Analytical and Environmental Sciences, National Tsing Hua University, 101, Sec 2, Kuang Fu Road, Hsinchu 30013, Taiwan; 4 Nanotechnology & Catalysis Research Centre, University of Malaya, Kuala Lumpur 50603, Malaysia

**Keywords:** biochar, photocatalysis, nickel nanoparticles, dye degradation

## Abstract

Photocatalysis has been recognized as a feasible method in water and wastewater treatment. Compared to other methods such as adsorption and chemical oxidation, the use of photocatalyst in the advanced oxidation processes gives benefits such as a longer lifetime of the catalyst and less consumable chemicals. Currently, explorations into low-cost, effective photocatalysts for organic contaminated water are being developed. Within this scheme, an easily separated photocatalyst with other functionality, such as high adsorption, is important. In this research, preparation of a magnetic nanocomposite photocatalyst based on agricultural waste, palm leaves biochar impregnated nickel nanoparticles (Ni/BC), was investigated. The nanocomposite was prepared by direct pyrolysis of palm leaves impregnated with nickel (II) chloride precursor. Furthermore, the physicochemical characterization of the material was performed by using an X-ray diffractometer (XRD), scanning electron microscopy-energy dispersive X-ray fluorescence (SEM-EDX), transmission electron microscopy (TEM), gas sorption analysis, X-ray photoelectron spectroscopy (XPS) and vibrating sample magnetometer (VSM). The photocatalytic activity of Ni/BC was evaluated for methyl violet (MV) photocatalytic oxidation. The results from XRD, XPS and TEM analyses identified single nickel nanoparticles dispersed on the biochar structure ranging from 30–50 nm in size. The dispersed nickel nanoparticles increased the BET specific surface area of biochar from 3.92 m^2^/g to 74.12 m^2^/g oxidation. High photocatalytic activity of the Ni/BC was exhibited by complete MV removal in 30 min for the concentration ranging from 10–80 mg/L. In addition, the Ni/BC showed stability in the pH range of 4–10 and reusability without any activity change until fifth usage. The separable photocatalyst is related to magnetism of about 13.7 emu/g. The results highlighted the role of biochar as effective support for Ni as photoactive material.

## 1. Introduction

Water is the most important and valuable natural resource for all human and biotic environments on earth, since it covers 70% of the earth’s surface. With developing anthropogenic and industrial activities, water pollution is a problem which is commonly faced today [1,2]. It is a widely recognized fact that pollution of water resources is a common problem. Significant attention is paid to water treatments including organic compounds and dye-contaminated water. Due to the hazardous potencies and toxicological effects of organic-polluted water, many techniques such as chemical oxidation, ozonization and electrocatalytic degradation have been developed. Referring to previous works on water and organic compound-contaminated water treatments, photocatalytic oxidation is one of the methods needed to be applied on an industrial scale [3,4]. 

The performance of this method, which does not need further procedures and a long lifetime for the photocatalyst material in the system, are the benefits, economically, compared to the adsorption method, for example. In addition, low-cost photocatalytic methods are being aggressively explored. The combination of high adsorption and easily separable photocatalyst have influenced the development of magnetic porous material [5,6]. Moreover, utilization of naturally occurring minerals and these abundant materials exhibit their potency for these needs. Within this scheme, biochar-based photocatalysts have proved their potential applicability as low-cost carbonaceous materials to support photoactive metal/metal oxide photocatalysts [7,8] The supportive adsorption mechanism of biochar provides acceleration for photocatalysis. 

Besides iron oxide magnetic materials, nickel nanoparticles (Ni NPs) are a magnetic material with optical properties, stability and durability for catalytic and photocatalytic applications [9,10,11]. In addition, the combinations of Ni NPs with other metal, metal oxides and biochar exhibited enhanced performance in catalytic efficiency [12]. Based on these backgrounds, in this research, a composite of magnetic Ni NPs supported on biochar was prepared and applied as photocatalyst material. Even though NiO-based nanocomposites have been widely reported as photocatalysts, to our knowledge the combination of Ni NPs in the biochar composite for photocatalysis purposes has not yet been reported and, particularly, the combination of photoactive with magnetic features could not be achieved by NiO NPs. Considering the huge amount of palm leaf as agricultural waste from palm plantation area in Indonesia and its carbon-rich characteristics, palm leaves were utilized as the raw material. For the photocatalytic activity test, methyl violet (MV) was selected as dye model due to its importance in textiles, printing, adhesive, ink, toner and other industries [13,14].

## 2. Results and Discussion

### 2.1. Physicochemical Characterization

**Figure 1:**
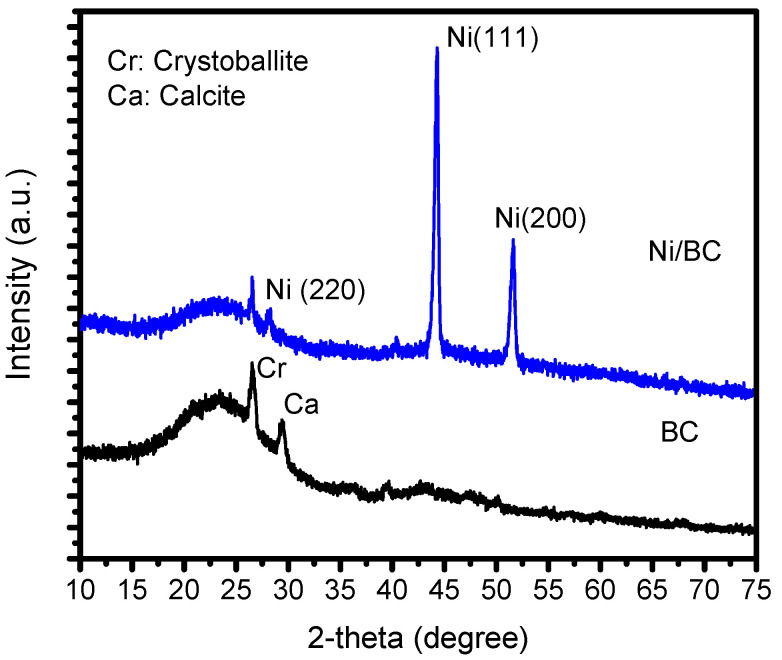
XRD patterns of BC and Ni/BC.

The XRD analysis of Ni/BC in comparison with BC is shown by the XRD pattern presented in Figure 1.

From the obtained diffractograms, it can be seen that different reflections appeared in Ni/BC with respect to the BC pattern. The diffractogram of Ni/BC exhibits specific peaks at 2θ of 27.5°, 44.8 and 52.3°, associated with 220, 111 and 200 reflections of a-FCC of single nickel nanoparticles (Ni), referring to JCPDS: 03-1051 [15,16,17]. In addition, a small broad peak ranging from 20°–30° is also identified similar to the broad peak in BC, implying the presence of a structure of aromatic layers (graphite 002) [18,19]. The broad peak expresses a small dimension of crystallites perpendicular to the aromatic layers as the characteristic of biochar structure. Particularly, the minor constituents of cristobalite and calcite also appear at 2θ of 26° and 28°, respectively. These miscellaneous inorganic components are generally found as the main constituent of agricultural waste [20,21]. The single nickel peakobtained, rather than another nickel oxide phase, reflected the success of the reducing mechanism by inert nitrogen flow in a pyrolysis system to the nickel ion precursor obtained via the impregnation method. The inert heating system procedure was similar to the argon-atmosphere heating regarding the magnetic single nickel nanoparticles reported in previous work [22,23,24]. Furthermore, the crystallite size (*D*) of the dispersed Ni NPs was determined by using the Scherer Equation (Equation (1)):

(1) $$D=\frac{K\lambda }{\beta \;cos\theta }$$

where *K* is reflection constant, *λ* is wavelength of XRD light, *β* is full width and half maximum (FWHM) of the reflections, and *θ* is the reflection angle. The calculated crystallite size is 23.6 nm. 

**Figure 2:**
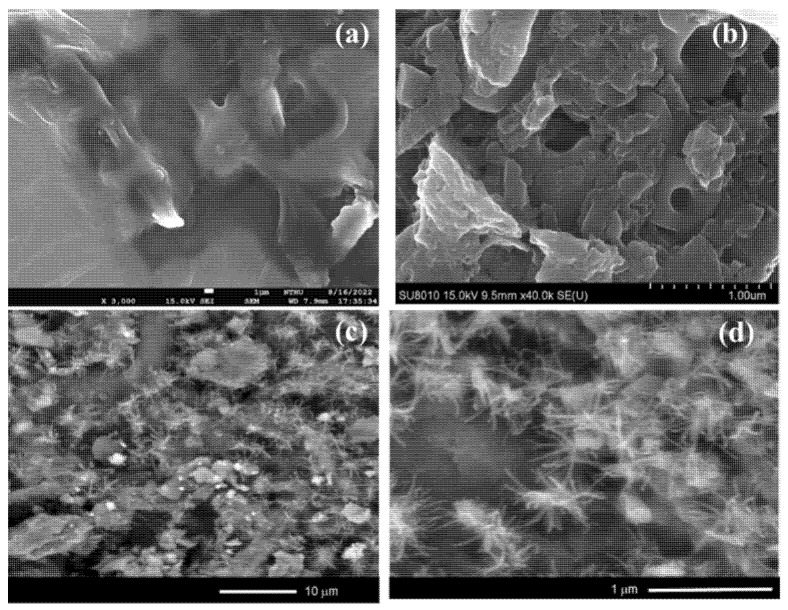
SEM images of (**a**,**b**) BC and (**c**,**d**) Ni/BC in different magnifications.

The dispersion of the Ni NPs in the Ni/BC nanocomposite is reflected by the change of surface morphology compared to the surface profile of BC analyzed by SEM, and the micrographs are depicted in Figure 2. BC shows a porous surface morphology which is characteristic of biochar; furthermore, the dispersed spots and nanowires on Ni/BC represent the dispersed Ni NPs on the surface. From previous studies, the nanowire form of the nanoparticles is attributed to the crystallite growth controlled by temperature and templating conditions. The pyrolytic condition of the carbon-rich material in the pyrolytic system may be attributed as influencing the aggregation of Ni NPs [25,26]. This is confirmed by the EDX analysis results presented in Table 1, suggesting an Ni amount of about 31.67 wt.% in Ni/BC. The higher Ni content in the Ni/BC compared to the set-up content of Ni (30 wt.%) could affect the weight loss of the cellulosic component during the pyrolysis process. In addition, the identified cristobalite and calcite from XRD analysis is in line with the obtained Si and Ca minerals as a minor component in the EDX analysis, respectively.

**Figure 3:**
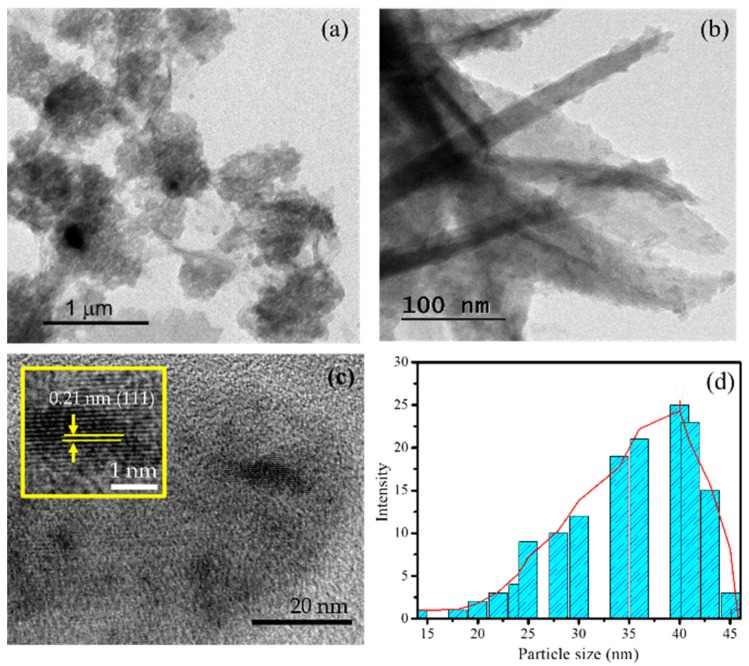
(**a**,**b**) TEM images of Ni/BC; (**c**) HRTEM image of Ni NPs; (**d**) Pore size distribution.

The occurrence of the Ni NPs in the Ni/BC nanocomposite is strengthened by the TEM and HRTEM images presented in Figure 3. Figure 3a shows the heterogeneous forms of the particles with a highlighted nanowires-like structure, in line and associated with the structures identified by the SEM image. In higher magnification (Figure 3b), the nanowires’ structures are clearly identified. Moreover, by HRTEM analysis, the fringes of Ni NPs are expressed with a distance of 0.21 nm (Figure 3c). These fringes space are associated with (111) reflections of Ni nanoparticles [27]. Based on the heterogeneous spherical particles, particles size distribution is as presented in Figure 3d, and it can be concluded that the particle size ranges from 30–50 nm.

**Figure 4:**
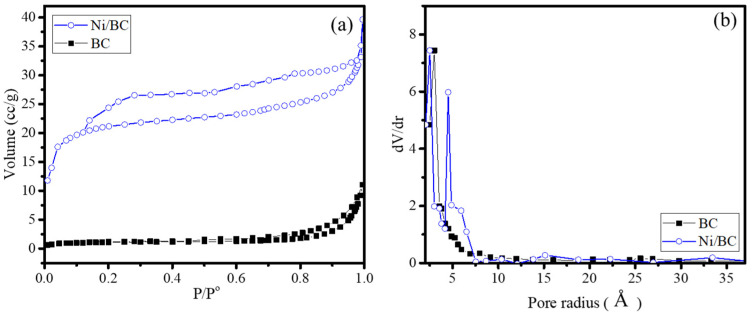
(**a**) Adsorption-desorption isotherm; (**b**) Pore size distribution of BC and Ni/BC.

The dispersion of Ni NPs in Ni/BC nanocomposite affects to the surface profiles as identified by the adsorption/desorption profile presented in Figure 4a and, based on the isotherm data, the calculated Brunair-Emmet-Teller (BET) specific surface area, pore volume and pore radius are listed in Table 2. 

From the isotherm patterns, it can be implied that there is a change in the isotherm shape from type III in BC sample into type IV in the Ni/BC sample. In addition, the hysteresis loop of the Ni/BC sample classified as type IV indicates that the pore shape is silt-pore [28]. This is relevant as the pore size distribution (Figure 4b) exhibits the formation of dominant micropore size at around 6 Å, besides the predominantly microporous structure in Ni/BC. Meanwhile, the BC expresses a microporous structure without any dominant pore size. The pore distribution also indicates that the formation of Ni nanoparticles aggregates on the surface, creating a surface area having the capability to adsorb the N_2_ adsorbate, which feature provides more of an adsorption site for enhancing the surface mechanism in catalysis or photocatalysis. This increased specific surface area with the presence of nickel precursor in biochar production is similar as that reported by previous works [29,30]. The existence of nickel salt during the pyrolysis process produces higher porosity due to the swelling capability of lignocellulosic decomposition. Moreover, the homogeneous distribution of nickel aggregate does not block the porosity. 

This confirms the influence of nickel salt, in line with the tendency towards a created porosity by the addition of nickel. 

**Figure 5:**
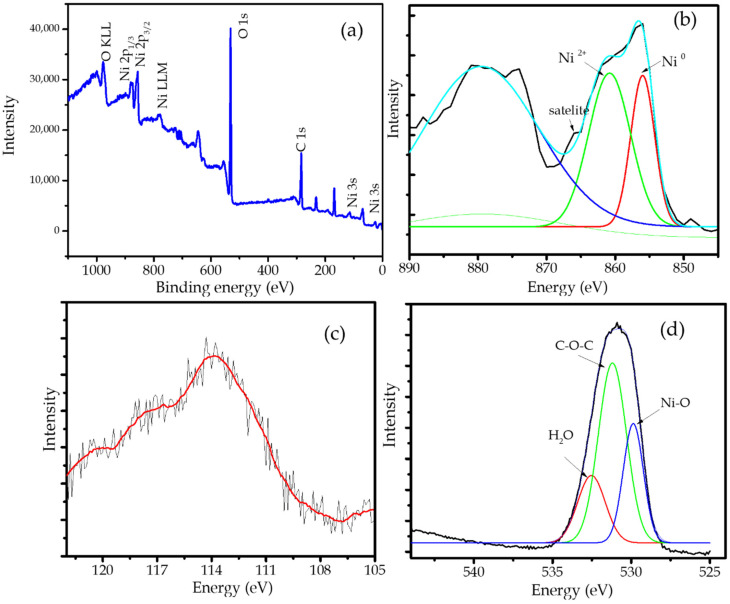
(**a**). Survey scan of Ni/BC; (**b**). Deconvolution of Ni 2p spectrum; (**c**). Ni 2s spectrum; (**d**). Deconvolution of O 1s spectrum.

The confirmation of Ni NPs in the nanocomposite is shown by the XPS spectrum presented in the survey scan (Figure 5a). The survey scan spectrum revealed peaks corresponding to C1*s*, O1*s* as the main component of biochar, while the presence of Ni NPs is identified by Ni2*p*, Ni3*s*, and Ni LLM spectra. The ionic state of Ni is justified by the intense Ni2*p* spectrum which exhibits two peaks at 856.5 and 873.2 eV. The deconvolution of the Ni spectrum (Figure 5b) revealed the occurrence peak at 853.3 eV as a Ni^0^ peak and the peak at 862.5 eV coming from Ni^2+^. The existing Ni^2+^ peak is aroused from the coordinated nickel with hydroxyl or oxygen functional group as support, and the presence of Ni on the surface site, which is also strengthened by the satellite’s peaks [31,32]. In addition, the 3s peak (Figure 5c) shows at 113.9 eV indicating the presence of Ni^0^. Further confirmation is also expressed by the O 1s spectrum in Figure 5d. The characteristic peaks at 528.5, 532.1, and 533.0 eV are assigned to the Ni-O, C C–O–C, and H_2_O, respectively [33,34].

### 2.2. Photocatalytic Activity

**Figure 6:**
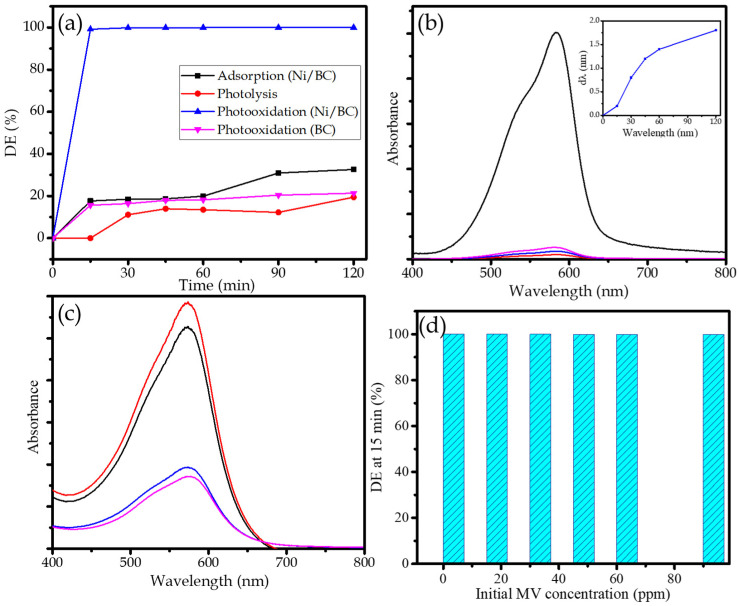
(**a**). Comparison on kinetics of adsorption, photolysis and photooxidation over BC and Ni/BC; (**b**). The change of MV spectrum by photooxidation; (**c**). The change of MV spectrum by adsorption; (**d**). Effect of initial MV concentration on DE of photooxidation.

The photocatalytic activity of Ni/BC was evaluated via photocatalytic oxidation (photooxidation) of MV. The kinetics of photooxidation over Ni/BC in comparison with photooxidation over BC, photolysis and the adsorption treatment of MV are presented in Figure 6. From the kinetics plot of photooxidation over Ni/BC, it is seen that photooxidation produces fast MV removal, in which almost 99% of DE was reached by the treatment after 15 min. In comparison with the controlled processes, DE of 62 and 20% are achieved by the adsorption and the photolytic process in 1 h, respectively. The compared plots represent the role of the photocatalyst along with UV light as source of photons to accelerate the oxidation of MV. Even though there was MV removal under the presence of the photocatalyst without light in the adsorption mechanism, the removal is still lower compared to photooxidation and, moreover, less removal exhibited by the presence of H_2_O_2_ with UV light illumination in the photolysis treatment. In addition, the kinetics of photooxidation over BC seems to be similar to the kinetics of adsorption over Ni/BC. These data imply that the accelerated MV removal occurred due to the combination of the adsorption mechanism from the provided specific surface area and the pore volume of Ni/BC with the presence of H_2_O_2_ and a photon source. The comparison also express that Ni NPs as the responsible photoactive sites to conduct the photooxidation mechanism are significantly measured by the comparison of photooxidation by BC and Ni/BC. The degradation is tremendously expressed by Ni/BC, but does not appear over BC. The slightly higher removal by photooxidation (BC) represents that the removal mechanism over BC occurred due to adsorption and photolysis in the presence of H_2_O_2_, but there is no sufficient propagation to oxidize MV. 

The rapid heating of Ni NPs while absorbing photons of the incident radiation generates electrons. Furthermore, the electrons combine with O_2_ and H_2_O as solvent to create ∙OH and O_2_^−^ species. The propagation leads to the production of some radicals and super radicals by the interaction with peroxide and dye molecule [35,36]. The degradation mechanism is proven by the change of MV spectrum by the photooxidation and adsorption mechanism (Figure 6b,c). The adsorption leads to the reducing absorbance values at the same wavelength (576 nm); meanwhile the photooxidation produces not only the reducing absorbance but also the shift in peak to the smaller wavelength, along with the increasing time of treatment (inset). This shift is identification of with the de-ethylation mechanism [37,38]. This means that the whole process causing MV removal is clearly photooxidation, causing the degradation of MV chemical structure rather than reducing concentration by the adsorption mechanism. 

The high effectiveness of Ni/BC for MV removal is represented by the maintained high values of DE at varied initial MV concentrations ranging from 10–80 ppm (Figure 6d). It is recognized that the DE reached more than 99.9% only at 15 min and a photocatalyst dosage of 0.25/250 mL. The detailed study of the kinetics of photooxidation at varied initial MV concentration was performed by approaching first-order and second order kinetics equations (Equations (2)–(4)):

(2) $$ln\frac{C_{t}}{C_{0}}=-k_{1}t$$

(3) $$\frac{1}{C_{t}}=k_{obs}t+\frac{1}{C_{0}}$$

where *C_t_* and *C*_0_ are concentrations of MV at time *t* and at the start, *k*_1_ is first-order kinetics constant, and *k*_2_ is second-order kinetics constant. The kinetics equation and R^2^ parameters are listed in Table 3. From the R^2^, it is conclusively obtained that the photooxidation reactions obey second order better than first order kinetics. 

These DE values are classified as highly efficient compared to other Ni-based photocatalyst usage in the same MV dye molecule photocatalytic oxidation reaction as presented in Table 4. The comparison demonstrated the excellent activity obtained by Ni/BC in this work as other Ni and NiO-based photocatalysts such as Ni-Ag bimetal, Ni/zeolite Y, Ni/SiO_2_, NiO NPs and Ni NPs expressed the DE at the range of 20–90% using longer time of treatment.

### 2.3. Effect of pH

**Figure 7:**
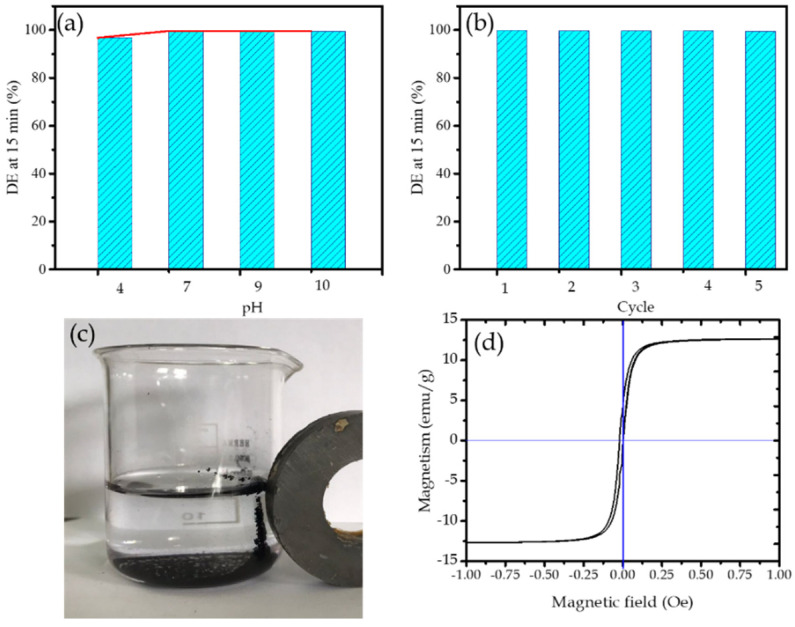
(**a**). Effect of pH on DE; (**b**). DE at varied cycles of Ni/BC usage in photooxidation; (**c**). Magnetic attraction of Ni/BC after usage; (**d**). VSM plot of magnetism of Ni/BC.

As in the mechanism in which the surface interaction between target molecule and photocatalyst is involved, it has been discussed that pH crucially influences the process’s and efficiency. Figure 7a shows the effect of pH on DE. From the plot, it can be concluded that the DE remains high (>99%) at all tested pH (4–10). The values suggest that, meanwhile and in particular, the adsorption of cationic species is extremely dependent on the pH of the solution and the oxidative mechanism conducted by the photoactive material plays a more important role. This means that Ni/BC is stable at all ranges of pH condition. However, in more detail, the DE at pH 7 is the highest value (99.80%) compared to other pH variations. This implies that the neutral surface facilitates the more intensive interaction between MV as molecule target with Ni/BC surface. The DE at pH 4 (96.88%) is the smallest value, representing that, in the acidic environment, less interaction of MV with Ni/BC occurred. This may be attributed to the competition of MV with H^+^ from the acidic environment causing less affinity of the surface for adsorption and undergoing of a photocatalysis reaction [6].

### 2.4. Reusability and Magnetic Susceptibility

Reusability of a photocatalyst is one of the important features regarding applicability at the industrial scale. The evaluation of Ni/BC reusability as photocatalyst is examined for five cycles of photooxidation experiments and the results are presented as a chart in Figure 7b. It is seen that, overall, for 1–5th cycle, DE values are maintained at above 99% without any significant change. This represents that the capability of Ni NPs as photoactive material in Ni/BC nanocomposite remains stable. The reusability of Ni/BC is also related to the easiness in separation after use, as expressed by Figure 7c. The magnetic attractivity of Ni/BC is confirmed by VSM plot in Figure 7d. The plot shows that Ni/BC expressed superparamagnetic characteristics with a saturation magnetization value (M*_s_*) of approximately 13.7 emu g^−1^. Ni/BC can be easily attracted by a magnetic field, suggesting easy separation and collection from the reaction system by using an external magnet.

**Figure 8:**
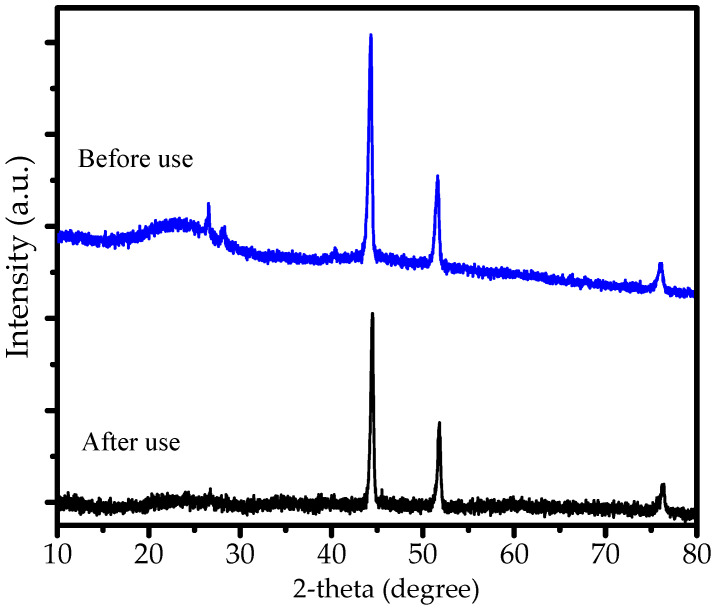
Comparison of XRD pattern of Ni/BC before and after use.

The stability of Ni/BC is confirmed by XRD pattern before and after use presented in Figure 8. As can be seen from the peaks, there is no change of Ni peaks observed, suggesting that there is no oxidation/reduction influencing the phase of Ni nanoparticles on the surface. 

In summary, from the characterization and photoactivity data of Ni/BC, it is reported that Ni/BC nanocomposite exhibited supportive physicochemical characters for photocatalysis mechanism. The high DE at a wide range of MV concentrations, as well as the reusability, are potential features for further development at a larger scale and for other organic compounds removal over photocatalytic oxidation.

## 3. Materials and Methods

### 3.1. Materials 

Nickel (II) chloride, H_2_O_2_ and methyl violet were purchased from Merck (Darmstadt, Germany). Palm leaves were obtained from West Kalimantan palm agricultural area. Gas N_2_ at ultra-high purity was supplied from PT Samator, Indonesia. 

### 3.2. Preparation Magnetic Ni NPs/Biochar (Ni/BC)

**Figure 9:**
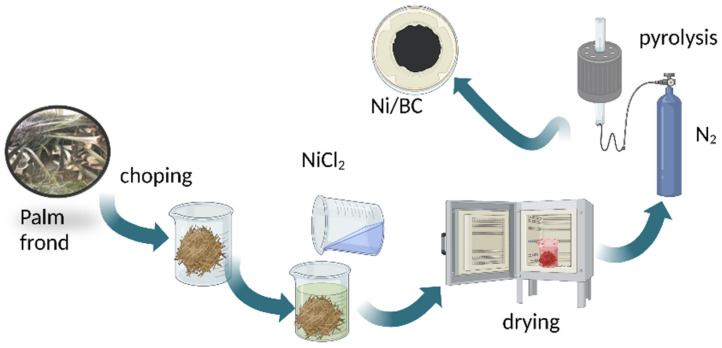
Schematic representation of Ni/BC preparation.

The Ni/BC sample was prepared by previously mixing the chopped palm leaves with NiCl_2_ solution at the set Ni concentration of 20 wt.% in the composite. The mixture was dried in the oven prior to pyrolysis at a temperature of 500 °C for 1 h under N_2_ gas flow. Figure 9 presents the scheme of Ni/BC preparation. 

### 3.3. Physicochemical Characterization

Relevant physicochemical characterizations consisting of XRD, SEM-EDX, TEM and VSM analyses were performed. A Bruker D8 DISCOVER diffractometer (Billerica, MA, USA) with Ni-filtered- Cu Kα radiation (40 kV and 30 mA) was utilized for XRD analysis. A JEM-7401 SEM instrument was employed for surface micrographic analysis; meanwhile, a Phenom-X instrument was utilized for EDX analysis. For TEM analysis, JEOL 2010F Field Emission instrument was used. TEM was operated at the applied voltage of 200 kV, along with monochromatic Al *K*_α_ radiation with a photon energy of 1486.6 ± 0.2 eV. The degassing of the sample prior analysis was at the pressure below 10^−8^ Pa for 4 h. The surface parameters consisting of specific surface area, pore volume, and pore radius determination were carried out on a NOVA 1200 gas sorption analyzer. X-ray photoelectron spectroscopy (XPS) analysis was conducted by using V.G. Scientific ESKALAB MKI microscope (Tokyo, Japan). For magnetism analysis, a vibration sample magnetometer (VSM)-BHV-5 (Tokyo, Japan) was employed.

### 3.4. Photocatalytic Activity Test

The photocatalytic activity of Ni/BC was carried out in the MV photocatalytic oxidation. For each experiment, about 0.25 g of Ni/BC powder was added into 250 mL of MV solution. The mixture was placed in a batch photocatalytic reactor equipped with a UV lamp. The reactor consists of 500 mL water-jacketed flask in a flask, and the center of the glass chamber, a Philips UV lamp (20 watt) with a wavelength of 296 nm and light intensity of 39.99 MW/Cm^2^ is placed. The sampling was performed sequentially by pipetting the of treated solution over a period of time. The effectivity of photocatalytic treatment was measured as the degradation efficiency (*DE*) which was calculated by using the following equation (Equation (4)):

(4) $$DE\left(\%\right)=\frac{RhB_{0}-RhB_{t}}{RhB_{0}}\;\times \;100\;$$

*RhB*_0_ and *RhB_t_* are the *RhB* initial concentration and concentration at time of *t*.

In order to evaluate the significantly different mechanisms of degradation, the experiments for adsorption and photolysis were also performed. In detail, the adsorption experiment was a similar procedure but without H_2_O_2_ addition and light exposure, while for the photolysis treatment the system is without a photocatalyst. For photocatalytic oxidation treatment, 3% of H_2_O_2_ solution was added into the mixture followed by UV exposure.

## 4. Conclusions

In this study, the magnetic nanocomposite of Ni/BC has been successfully prepared by using palm leaves biochar. From the XRD, SEM, TEM, HRTEM analyses, it is found that a single Ni NPs dispersed onto biochar with nanowires structure is expressed by the nanocomposite. The increased specific surface area resulted from the dispersion along with magnetism of 13.7 emu/g and band gap energy of 2.7 eV. The nanocomposite was found to have strong activity as photocatalyst in MV photocatalytic oxidation. The obtained DE values are above 99% for the MV range of 10–80 ppm of treatment for 30 min. In addition, reusability of Ni/BC is expressed by the maintained DE values until fifth cycle of usage.

## Figures and Tables

**Table 1:** Elemental analysis of BC and Ni/BC from EDX analysis.

Element	Percentage in (wt. %)	
	BC	Ni/BC
O	40.81	15.81
C	18.17	32.86
Al	12.42	1.82
Si	12.07	6.56
K	8.73	0.97
Fe	6.81	1.96
Ca	0.98	1.04
Ni	n.d.	31.67

**Table 2:** BET specific surface area, pore volume and pore radius of BC and Ni/BC.

Parameter	BC	Ni/BC
Specific surface area (m^2^/g)	3.92	74.12
Pore volume (cc/g)	1.64 × 10^−3^	2.89 × 10^−3^
Pore radius (Å)	31.98	6.79

**Table 3:** Kinetics parameter and equation.

MV Concentration (ppm)	First-Order Kinetics		Second-Order Kinetics	
	R^2^	Kinetics Equation	R^2^	Kinetics Equation
10	0.898	*ln C* = 0.307 *t* + 0.305	0.999	*1/C* = 66.66 *t* + 0.167
20	0.901	*ln C* = −0.060 *t* + 2.324	0.974	*1/C* = 4.282 *t* + 49.639
50	0.869	*ln C* = −0.053 *t* + 3.695	0.990	*1/C* = 0.619 *t −* 4.390
70	0.861	*ln C* = −0.104 *t* + 2.635	0.955	*1/C* = 0.255 *t −* 1.534
80	0.867	*ln C* = −0.080 *t* + 3.029	0.957	*1/C* = 0.042 *t +* 0.064

**Table 4:** Comparison on activity of Ni and NiO-based photocatalyst in MV photooxidation.

Photocatalyst	DE	Remark	
NiO NPs	28	Photocatalysis reaction obeys pseudo-first order kinetics	[39]
NiO-Ag bimetal	32	Photocatalysis reaction obeys pseudo-first order kinetics at MV concentration of 1 × 10^−3^ M at neutral pH	[39]
Activated carbon-supported NiS/CoS	60–70	Degradation efficiency is ranging at 56–78% depending on Ni and Co composition at degradation time of 90 min	[40]
Ni NPs	45	Ni NPs was synthesized using polyvinyl pyrrolidone (PVP), stabilizer, the reaction was conducted under UV light for 40 min	[41]
Ni/Zeolite Y	94	Photocatalysis reaction was conducted for 240 min	[42]
NiO/SiO_2_	20	Photooxidation was conducted for 21 min	[43]
NiO	50	Photooxidation was conducted for 21 min	[43]
Ni/BC	>99.5	Photooxidation efficiency was obtained for MV initial concentration of 10–80 ppm conducted for 30 min	This work

## Data Availability

Not applicable.
